# Chitosan‐Based Nanoparticles for Twist1 Knockdown in 4T1 Cells

**DOI:** 10.1002/mabi.202400627

**Published:** 2025-04-10

**Authors:** Asim Mushtaq, Li Li, Anitha A, Lisbeth Grøndahl

**Affiliations:** ^1^ School of Chemistry and Molecular Biosciences The University of Queensland Cooper Road Brisbane Queensland 4072 Australia; ^2^ Australian Institute for Bioengineering and Nanotechnology The University of Queensland Corner of College and Cooper Road Brisbane Queensland 4072 Australia

**Keywords:** chitosan, nanomedicine, poly(ethylene glycol), SiRNA delivery, twist1

## Abstract

Bone metastasized breast cancer reduces the quality of life and median survival. Targeted delivery of twist1‐siRNA using nanoparticles (NPs) is a promising strategy to overcome current limitations in treating such metastatic breast cancers. This research evaluates two types of chitosan (CHI)‐based NPs for the delivery of twist1‐siRNA. Alendronate conjugated PEG functionalized chitosan (ALD‐PEG‐CHI) NPs are developed for active targeting while PEG functionalized CHI (mPEG‐CHI) NPs are fabricated for passive targeting. The size of twist1‐siRNA‐loaded NPs is below 70 nm and the zeta potential is near neutral for both types of NPs. Based on gel retardation assay, complete encapsulation of twist1‐siRNA is achieved in both NP systems. The ALD‐PEG‐CHI‐siRNA and mPEG‐CHI‐siRNA NPs display serum protection for 6 and 4 h, respectively, compared to the immediate degradation of naked twist1‐siRNA. The NPs can knockdown twist1 in 4T1 cells as demonstrated through protein expression as well as by phenotypic change in directional cell migration by wound healing assay. Overall, these in vitro results illustrate the potential of the NPs as an effective therapeutic system for bone metastasized breast cancer.

## Graphical abstract

Modified chitosan nanoparticles have the potential for passive and active siRNA delivery to different tumors. This study evaluates twist1‐siRNA‐loaded pegylated chitosan and alendronate‐conjugated pegylated chitosan nanoparticles for twist1 gene silencing in 4T1 cells. Twist1 gene silencing is confirmed through delayed wound healing and reduced twist1 protein expression. This shows the potential of these nanoparticles for bone metastasized breast cancer.

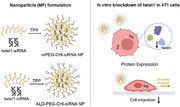

## 1 Introduction

Breast cancer is one of the main causes of death in women globally.^[^ ^1^, ^2^ ^]^ It can metastasize to different organs and tissues, where bone tissue is one of the main metastasis sites. Such metastasis of breast cancer at bone tissue significantly affects both the quality of life and survival prognosis of patients.^[^ ^3^ ^]^ Recently, gene therapy using siRNA for the treatment of different cancers has gained increasing attention due to its low cytotoxicity and anticancer activity.^[^ ^4^, ^5^ ^]^ A challenge for the delivery of siRNA is the different extracellular and intracellular hurdles that restrict the application of siRNA in clinics. Naked siRNA exhibits unsatisfactory stability and poor pharmacokinetic behavior due to rapid degradation by the endo or exonucleases in the body after systemic administration.^[^ ^6^ ^]^ Therefore, in recent years, nanomedicine has emerged as a potential solution for the delivery of siRNA in cancer therapy.

A suitable carrier system that is able to enhance the stability of siRNA in different biological fluids, protect it from nucleases, and improve bioavailability^[^ ^7^, ^8^ ^]^ is the biodegradable biopolymer chitosan (CHI).^[^ ^5^, ^9^ ^]^ It readily forms polyelectrolyte complexes with siRNA due to their opposite charge. Although PEGylation of polymers or nanoparticles (NPs) can potentially generate an immune response, PEGylation remains an important strategy used to improve the chemical and biophysical properties of NPs.^[^ ^10^, ^11^ ^]^ The presence of PEG molecules enhances hydrophilicity and provides steric stabilization to NPs,^[^ ^12^ ^]^ resulting in PEGylated CHI particles displaying passive targeting.^[^ ^5^, ^13^, ^14^ ^]^ To achieve active targeting of bone cancer, the functionalization of NPs with the bisphosphonate alendronate (ALD) has shown great promise.^[^ ^5^, ^13^, ^14^ ^]^

Twist1 is identified to play roles in tumor initiation, migration, invasion, and metastasis.^[^ ^15^, ^16^ ^]^ It activates or represses its target genes by binding to E‐box elements in the form of homo‐ or hetero‐dimers.^[^ ^17^ ^]^ It is expressed in different cancers such as liver, breast, gastric, and prostate, and its expression results in metastatic and invasive phenotypes.^[^ ^17^ ^]^ Moreover, its overexpression results in epithelial‐mesenchymal transition (EMT), a major process in metastatic cancers, and it also facilitates cancer stem cell formation, which results in tumorigenesis.^[^ ^18^, ^19^ ^]^ Recent research has demonstrated that twist1 accelerates bone metastasis in breast cancer through a microRNA‐10b (miR‐10b) dependent mechanism, which results in increased tumor burden and bone damage.^[^ ^20^ ^]^ It is found that twist1‐dependent seeding and colonization of breast cancer at bone tissue is dependent on miR‐10b and CD44 expression.^[^ ^20^, ^21^, ^22^ ^]^ In the case of breast cancer, twist1 overexpression is associated with the downregulation of estrogen receptor α, resulting in reduced sensitivity to different hormone therapies.^[^ ^23^, ^24^ ^]^ Moreover, twist1 overexpression is associated with resistance to commonly used chemotherapeutics in different cancers,^[^ ^25^, ^26^ ^]^ and while twist1 expression is rare in healthy adult tissues, it is overexpressed in many cancers.^[^ ^27^ ^]^ Gene silencing of twist1 is, therefore, an attractive means to mitigate metastasizes with minimal side effects on healthy tissues.

Only a few studies have explored the design and evaluation of delivery systems for the delivery of twist1‐siRNA. For a dendrimer‐based twist siRNA delivery system, twist1 knockdown was observed through reduced migration and invasion of breast cancer cells.^[^ ^28^ ^]^ Silica NPs were found to reduce the tumor size after twist1 knockdown^[^ ^29^ ^]^ and when co‐administered with cisplatin, twist1‐siRNA delivered by silica NPs displayed a reduction in disseminated tumors.^[^ ^30^ ^]^ Similarly, twist siRNA hyaluronic acid‐based NPs co‐administered with cisplatin reduced the tumor burden in ovarian cancer.^[^ ^31^ ^]^ Based on this limited but encouraging work, the current study explores the use of modified CHI‐based twist1‐siRNA delivery systems to evaluate the twist1 knockdown in MDA‐MB‐231 and 4T1 breast cancer cell lines. Specifically, twist1‐siRNA is encapsulated in two types of CHI‐based NPs, PEGylated CHI NPs (produced from PEG functionalised CHI, mPEG‐CHI) and PEGylated CHI NPs carrying the bone‐targeting ligand ALD (produced from alendronate conjugated PEG functionalised CHI, ALD‐PEG‐CHI). The former NPs were fabricated for passive targeting and the latter for active targeting. These NPs are evaluated for their in vitro serum protection and cytotoxicity. The twist1 knockdown was furthermore evaluated by protein expression and by phenotypic change in directional cell migration by wound healing assay.

## 2 Results and Discussion

### 2.1 NP Fabrication and Characterization

**Scheme 1:**
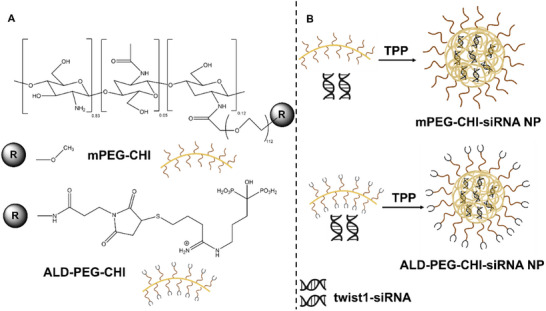
A) Chemical structures of mPEG‐CHI and ALD‐PEG‐CHI; B) Schematic illustration of the fabrication of twist1‐siRNA NPs prepared from mPEG‐CHI and ALD‐PEG‐CHI, respectively. Part of this figure was generated using BioRender.

The twist1‐siRNA loaded NPs were fabricated using two different PEGylated CHI‐based polymers, mPEG‐CHI^[^ ^32^ ^]^ or ALD‐PEG‐CHI,^[^ ^33^ ^]^ where the latter carry the bone‐targeting ligand ALD. This research builds on our previous work evaluating these NP systems encapsulating cell death‐siRNA and curcumin, where the hydroxyapatite‐seeking ability of ALD‐PEG‐CHI NPs was demonstrated.^[^ ^34^ ^]^ In the current study, both polymers had a degree of substitution of 12%, and their chemical structures are included in **Scheme** **1**.

**Figure 1:**
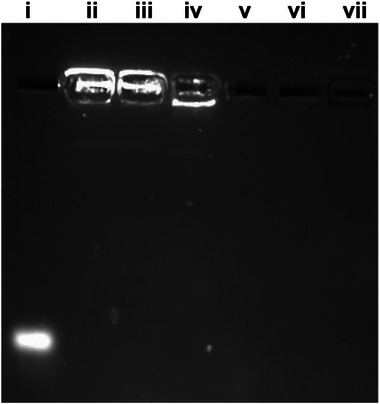
Gel retardation assay of siRNA in CHI, mPEG‐CHI, and ALD‐PEG‐CHI NPs systems along with the free siRNA in the filtrate (running buffer of pH 7.2–7.4). Labels represent i) naked siRNA, ii) CHI‐siRNA NPs, iii) mPEG‐CHI‐siRNA NPs, iv) ALD‐PEG‐CHI‐siRNA NPs, v) CHI NP filtrate, vi) mPEG‐CHI NP filtrate, vii) ALD‐PEG‐CHI NP filtrate. The labels ii to iv indicate the retention of siRNA‐loaded NPs in the wells and no band of free siRNA, while the labels v to vii show no band for filtrate in the wells and no free siRNA band. Collectively, this confirms maximum encapsulation of siRNA.

A previous study exploring CHI as a siRNA vector found that NPs fabricated with the use of the crosslinker tripolyphosphate (TPP) outperformed those prepared without TPP.^[^ ^35^ ^]^ A study comparing different crosslinkers (TPP, dextran sulfate (DS), and poly‐D‐glutamic acid (PGA)) found CHI NPs crosslinked with TPP were a more effective siRNA vector.^[^ ^36^ ^]^ The current study, therefore, used TPP as a crosslinker for the fabrication of twist1‐siRNA loaded NPs with a 3:1 and 5:1 polymer:TPP mole ratio for the fabrication of mPEG‐CHI and ALD‐PEG‐CHI NPs, respectively, as previously reported^[^ ^34^ ^]^ and illustrated in Scheme 1. As shown in **Figure** **1**, twist1‐siRNA has been completely encapsulated into mPEG‐CHI and ALD‐PEG‐CHI NPs without any leakage as evident from gel electrophoresis. Lanes iii and iv with the mPEG‐CHI and ALD‐PEG‐CHI NP samples display a clear band of twist1‐siRNA while no twist1‐siRNA can be detected in the filtrate from the NP samples (lanes vi and vii). Particles produced from unmodified CHI served as a positive control and displayed similar trends (lanes ii and v). This indicates an encapsulation efficiency of twist1‐siRNA in the NPs of near 100%.

**Figure 2:**
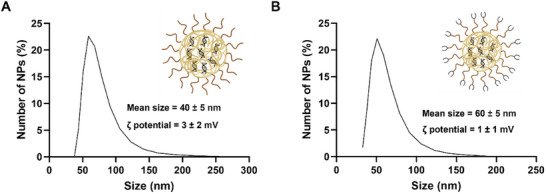
Size distribution data of A) mPEG‐CHI‐siRNA NPs, B) ALD‐PEG‐CHI‐siRNA NPs by number weighted distribution using DLS. *n* = 3. Errors represent the standard deviation. The ζ‐potential distribution plots are provided in Figure S1 (Supporting Information). Part of this figure was generated using BioRender.

The size and zeta potential of the CHI‐based NPs were evaluated using DLS, and the data are shown in **Figure** **2**A,B with the ζ‐potential distribution plots provided in Figure S1 (Supporting Information). The mean particle size for the mPEG‐CHI‐siRNA NPs was 40 ± 5 nm, while a value of 60 ± 5 nm was observed for the ALD‐PEG‐CHI‐siRNA NPs. Although the ALD‐conjugation caused a minor increase in particle size, this moderate size difference is anticipated to exert only minimal influence on biodistribution profiles. Previous literature indicates that NPs within the range of ≈20–100 nm maintain efficient cellular uptake and favorable biodistribution characteristics, supporting effective endocytosis and avoiding lysosomal uptake while also sustaining systemic circulation.^[^ ^5^, ^37^, ^38^, ^39^, ^40^, ^41^ ^]^

The ζ‐potential of the mPEG‐CHI‐siRNA and ALD‐PEG‐CHI‐siRNA NPs was +3 ± 2 and +1 ± 1 mV, respectively. This indicates that the use of polymers with a degree of 12% PEG substitution of CHI is a good compromise between retaining sufficient cationic groups for twist1‐siRNA encapsulation and constructing NPs with a near‐neutral charge. Similar NP properties were observed in previous studies when using PEG‐CHI for the encapsulation of siRNA,^[^ ^4^, ^34^ ^]^ while CHI‐based NPs of other studies retained a positive charge (ζ‐potential > 10 mV).^[^ ^42^ ^]^ In the current study, the presence of the bone‐targeting ligand ALD did not affect the ζ‐potential of the NPs, however, the size was significantly larger than NPs fabricated using mPEG‐CHI and this is attributed to the expected difference in swelling behavior of the NPs.^[^ ^34^ ^]^

### 2.2 Cytotoxicity of NP Systems

**Figure 3:**
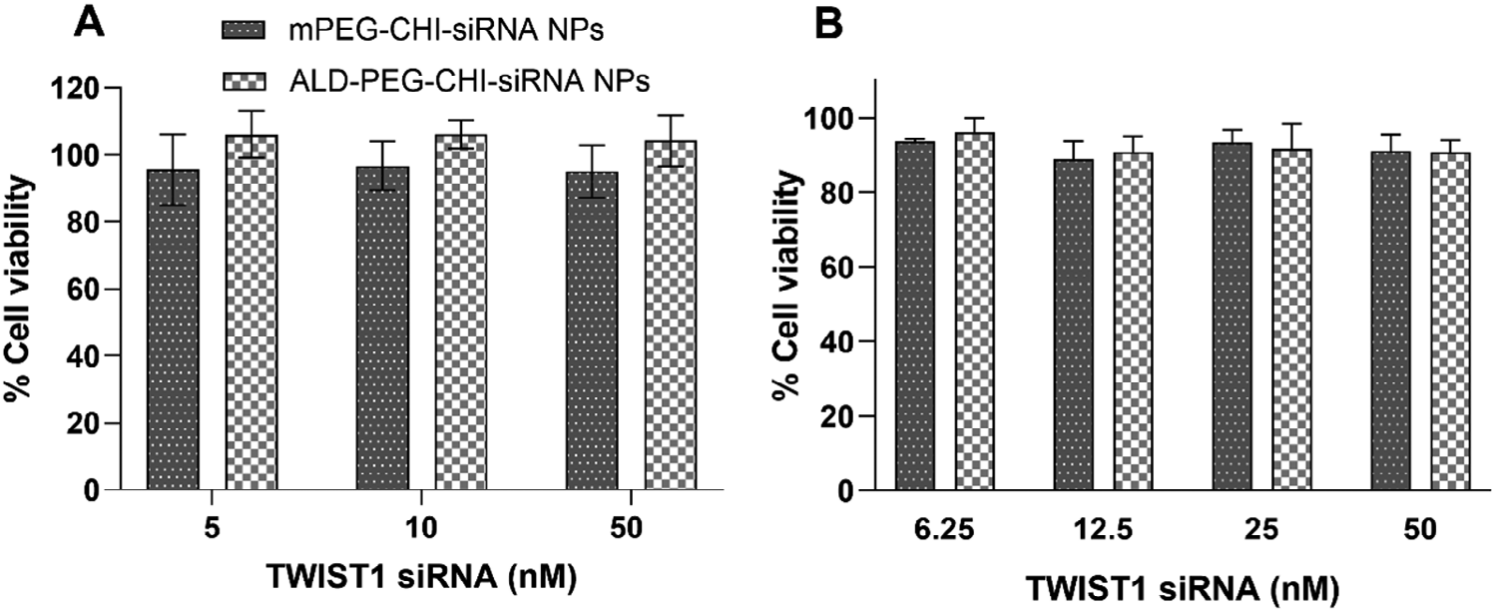
Cytotoxicity (MTT assay) of twist1‐siRNA loaded mPEG‐CHI‐siRNA and ALD‐PEG‐CHI‐siRNA NPs against A) 4T1 cells and B) MDA‐MB‐231 cells for 48 h. Seeding density = 3000 cells/well in a 96‐well plate, *n* = 3.

Two metastatic breast cancer cell lines MDA‐MB‐231 and 4T1 were used to evaluate twist1 knockdown by twist1‐RNA.^[^ ^43^, ^44^, ^45^, ^46^ ^]^ The mPEG‐CHI and ALD‐PEG‐CHI polymers as well as their NPs have previously been shown to be non‐cytotoxic against MDA‐MB‐231 cells.^[^ ^32^, ^34^ ^]^ mPEG‐CHI‐siRNA and ALD‐PEG‐CHI‐siRNA NPs were evaluated against MDA‐MB‐231 and 4T1 cells after 48 h incubation and found to be non‐cytotoxic in the concentration range of 5–50 nm equivalent twist1‐siRNA and 2–20 µg mL^−1^ equivalent polymer against 4T1 cells (**Figure** **3**A) and in the concentration range of 6.25–50 nm equivalent twist1‐siRNA against MDA‐MB‐231 cells (Figure 3B). These results agree with a previous study evaluating the cytotoxicity of twist1‐siRNA‐loaded mesoporous silica NPs against MDA‐MB‐435S cells which likewise found it to be non‐cytotoxic.^[^ ^29^ ^]^

### 2.3 Serum Protection of Twist1‐siRNA

**Figure 4:**
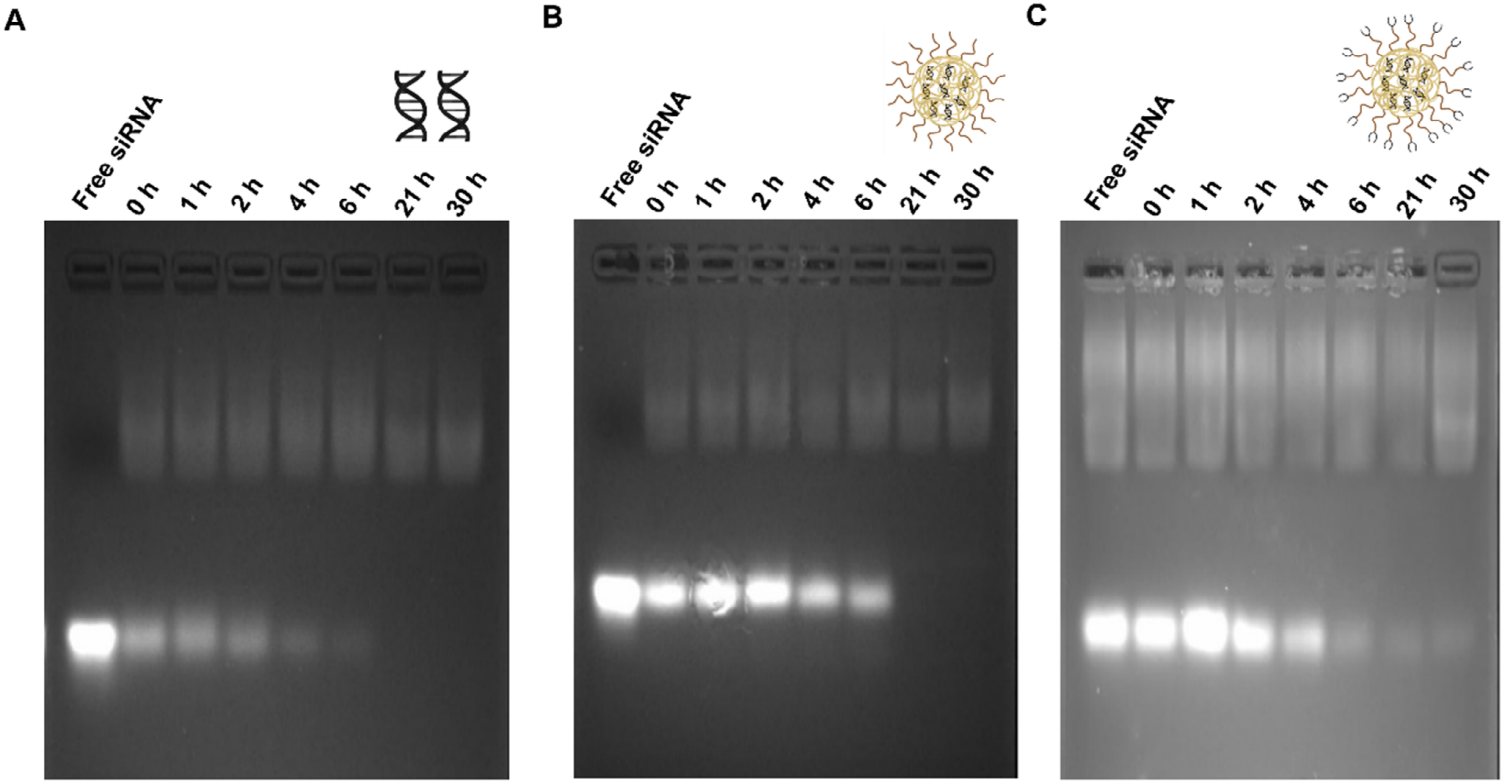
Electrophoretic mobility of A) control naked siRNA B) mPEG‐CHI‐siRNA NPs C) ALD‐PEG‐CHI‐siRNA NPs after incubating with cell culture medium DMEM containing 50% FBS. The same amount of free siRNA was used as a control. Samples were collected at different time points and stored at −20 °C before running them on the gel (running buffer pH 8.3). The faint bands of naked siRNA from 0 h timepoint show rapid degradation of siRNA in the presence of 50% FBS, while mPEG‐CHI‐siRNA NPs and ALD‐PEG‐CHI‐siRNA NPs protected the siRNA form serum nucleases up to 6 and 4 h, respectively. The presence of very faint or no bands at some time points is due to the full degradation of released siRNA. The faint bands toward the top of the gel relate to components from FBS. Part of this figure was generated using BioRender.

The ability of a nano‐therapeutic system to protect the payload against nucleases is an important aspect of delivering the nucleic acid moieties at the targeted site. To evaluate this, the serum protection of twist1‐siRNA by encapsulation in CHI‐based NPs was investigated and the data is shown in **Figure** **4**. The naked twist1‐siRNA was degraded immediately after mixing with fetal bovine serum (FBS) as shown in Figure 4A in agreement with previous studies.^[^ ^35^, ^36^ ^]^ In contrast, twist1‐siRNA encapsulated in the mPEG‐CHI and ALD‐PEG‐CHI NPs showed enhanced serum stability as indicated by prominent bands (Figure 4B,C). For the mPEG‐CHI‐siRNA NPs, twist1‐siRNA was protected from nucleases for 6 h (Figure 4B), while for the ALD‐PEG‐CHI‐siRNA NPs protection was sustained for up to 4 h (Figure 4C). The difference observed in the serum protection of twist1‐siRNA by the two systems correlates with the expected difference in their swelling behavior.

A previous study using PEG‐CHI NPs likewise observed enhanced serum protection by the NP system.^[^ ^47^ ^]^ Based on previous in vivo studies, a range of different NPs rapidly distribute to various organs (within 10 min in mice)^[^ ^48^ ^]^ and PEGylated gold NPs of 50 nm have been demonstrated to accumulate in tumor endothelial cells of solid tumors within 0–85 min.^[^ ^49^ ^]^ This indicates that if the siRNA payload is protected from serum nucleases for 2 h or more, the PEGylated NPs can reach the target cells and potentially be endocytosed within that time. Thus, the serum protection observed for the NPs of the current study of 4–6 h is potentially sufficient for the efficient delivery of twist1‐siRNA in vivo.

### 2.4 Twist1‐siRNA Mediated Twist1 Knockdown Evaluated by Protein Expression

**Figure 5:**
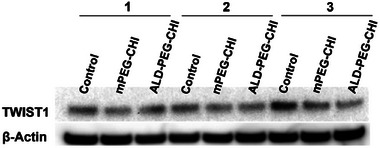
Western blot analysis of 4T1 cells. Cells were treated with twist1‐siRNA‐loaded NPs for 24 h. 1, 2, and 3 indicate repeats of the experiment (*n* = 3). Control represents untreated cells.

In order to mitigate the adverse effects of twist1 on cancer metastasis and chemotherapeutic resistance in cancers, twist1 silencing using twist1‐siRNA is a potentially attractive solution. Twist1 knockdown efficiency was determined by protein expression in 4T1 and MDA‐MB‐231 via western blot analysis. As displayed in Figure S2 (Supporting Information), twist1 expression was observed in the 4T1 but not in MDA‐MB‐231 cells. These findings are in agreement with Croset et al.^[^ ^20^ ^]^ and Sieuwerts et al.^[^ ^50^ ^]^ while other studies indicated the effective twist1 expression in MDA‐MB‐231 cells.^[^ ^51^, ^52^ ^]^ The twist1 knockdown of twist1‐siRNA delivered by mPEG‐CHI‐siRNA and ALD‐PEG‐CHI‐siRNA NPs was therefore evaluated only in 4T1 cells. As shown in **Figure** **5**, all samples displayed high expression of β‐actin (housekeeping protein), and twist1 protein levels were consistently lower for 4T1 cells treated with the mPEG‐CHI‐siRNA or ALD‐PEG‐CHI‐siRNA NPs compared to the control, suggesting that the twist1‐siRNA loaded NPs were endocytosed and caused inhibition of the twist1 gene inside the cells.

### 2.5 Twist1‐siRNA Mediated Twist1 Knockdown Evaluated from Phenotypic Change

The twist1‐siRNA knockdown was furthermore evaluated by phenotypic change in directional MDA‐MB‐231 and 4T1 cell migration by an in vitro wound healing assay which is a simple and well‐established method to evaluate the directional migratory potential of cells after treatment with different drugs.^[^ ^53^ ^]^ The data on the effect of twist1‐loaded NPs on MDA‐MB‐231 cells is displayed in Figure S3 (Supporting Information). It can be observed that there is no significant difference in the %wound closure in the absence or presence of the twist1‐loaded NP systems. This is in agreement with the lack of twist1 knockdown efficiency shown in Figure S2 (Supporting Information) and supports the lack of effective twist1 expression in MDA‐MB‐231 cells.

**Figure 6:**
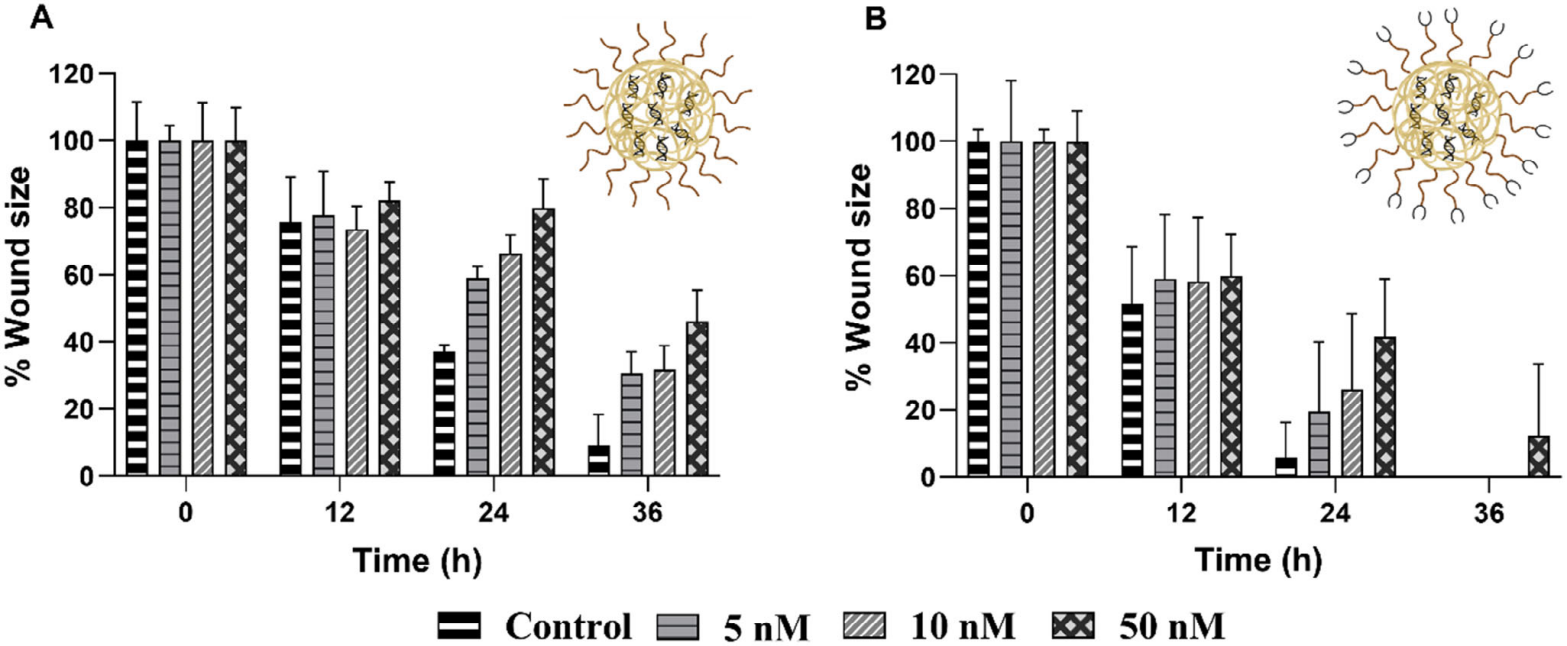
Wound healing effect of A) mPEG‐CHI‐siRNA NPs (seeding density = 2.8 × 10^4^ cells cm^−2^, *n* = 3) and B) ALD‐PEG‐CHI‐siRNA NPs (seeding density = 3.3 × 10^4^ cells cm^−2^, *n* = 3) against 4T1 cells. Part of this figure was generated using BioRender.

The data on the effect of twist1‐siRNA‐loaded NPs on delaying cell migration for the 4T1 cells are displayed in **Figure** **6**, while examples of the images used to generate this data are displayed in Figures S4 and S5 (Supporting Information). It was found that in this cell line, the twist1‐siRNA‐loaded NPs delayed cell migration. After 24 h of administration of 5, 10, and 50 nm equivalent twist1‐siRNA from mPEG‐CHI‐siRNA NPs (Figure 6A), 59, 66, and 80% of the remaining wound area was observed, respectively, compared to 37% remaining wound area of the control. For ALD‐PEG‐CHI‐siRNA NPs (Figure 6B), 19, 26, and 42% remaining wound area was observed compared to 6% of the control after 24 h. There was no significant difference in cell migration with different concentrations of twist1‐siRNA, however, a dose of 50 nm twist1‐siRNA resulted in the slowest cell migration by both mPEG‐CHI‐siRNA and ALD‐PEG‐CHI‐siRNA NPs compared to the control (*p*‐value <0.05). The migration degree of mPEG‐CHI and ALD‐PEG‐CHI NPs cannot be compared due to the different cell densities used in these experiments.

Overall, reduced expression of twist1 and phenotypic change of delayed cell migration indicated twist1‐siRNA has been effectively delivered into the 4T1 cells and decreasing twist1 expression in the cells consistent with previous work.^[^ ^28^, ^29^ ^]^ Chitosan and its derivatives have diverse properties which make them suitable candidates for formulation of drug delivery systems. A recent review paper on the use of chitosan in cancer therapy highlighted its dual role as a therapeutic agent and nano‐drug delivery system.^[^ ^54^ ^]^ Another promising aspect of previous work is the ability to reverse chemoresistance with the co‐delivery of twist1‐siRNA and cisplatin.^[^ ^30^, ^31^ ^]^ In our previous in vitro study^[^ ^34^ ^]^ we likewise demonstrated successfully enhanced synergistic effects for mPEG‐CHI NPs and additive effects for ALD‐PEG‐CHI NPs when co‐administering siRNA and a chemotherapeutic drug and our recent review paper^[^ ^5^ ^]^ identified co‐administration as essential for therapeutic in vivo effects of CHI‐based NP systems. A recent study likewise demonstrated the need for co‐administration of two drugs to complete tumor regression without any mortality in a prostate cancer model of bone metastasis.^[^ ^55^ ^]^

The use of NP systems conjugated with ALD has previously been shown to accumulate in the tumor region.^[^ ^13^, ^14^, ^56^, ^57^ ^]^ The ALD‐PEG‐CHI NP system evaluated in the current study has previously demonstrated the in vitro bone‐seeking ability.^[^ ^34^ ^]^ Therefore, taking together our findings and previous studies indicate that the use of ALD‐PEG‐CHI NP to co‐deliver twist siRNA and a chemotherapeutic drug is a promising active targeting therapeutic strategy for bone metastasis, while the use of mPEG‐CHI NPs shows promise for passive targeting.

## 3 Conclusion

Two modified CHI polymers were explored for twist1 transfection. Both modified CHI polymers stably encapsulated twist1‐siRNA at physiological pH (N/P ratio of 50). The twist1‐siRNA stability in serum evaluated by serum protection assay by heparin displacement indicated quick degradation of naked siRNA, however, encapsulated siRNA was found stable up to 6 h. Twist1‐siRNA‐loaded mPEG‐CHI and ALD‐PEG‐CHI NPs showed gene knockdown effects based on twist1 protein levels in 4T1 cells. The phenotypic change of cell migration was also observed for 50 nm equivalent twist1‐siRNA as it caused delayed wound healing. This demonstrates the suitability of the two NP systems of the current study with sizes of 40 and 60 nm and zeta potentials of near neutral to have the ability to effectively deliver siRNA through cellular uptake by 4T1 cells. It was observed that the ALD‐PEG‐CHI NPs displayed a higher degree of swelling and hence lower serum protection compared to mPEG‐CHI NPs. Future work will modify this formulation to optimize the regulation of the release of siRNA. Overall, this study provided robust in vitro evidence demonstrating the efficacy of these NP systems in silencing the twist1 gene, a critical step required before advancing to more intricate and resource‐intensive in vivo evaluations. Future work will explore the in vivo potential of these CHI‐based NP systems which will include evaluation of the ability to reverse chemoresistance and seek bone tumors.

## 4 Experimental Section

### Materials

mPEG‐CHI (CHI conjugated with methoxyPEG, M_w_ 5000 Da) was synthesized from medical grade CHI (M_w_ 80–200 kDa, degree of acetylation (DA) 5% by NMR, obtained from Heppe Medical Chitosan) as reported previously.^[^ ^32^ ^]^ ALD‐PEG‐CHI (CHI conjugated with ALD‐PEG, M_w_ 5000 Da) and medical grade CHI as described previously.^[^ ^33^ ^]^ Both polymers with a 12% degree of substitution were used. MDA‐MB‐231 and 4T1 cells were obtained from ATCC (American Type Culture Collection). Twist1‐siRNA (sense: 5′ GGACAAGCUGAGCAAGAUU 3′, antisense: 5′ AAUCUUGCUCAGCUUGUCUU 3′) was purchased from integrated DNA technologies. The size and purity of this twist1‐siRNA were confirmed as shown in Figure S6 (Supporting Information). Anti‐Twist antibodies: TWIST(2C1A), actin antibody (C4), and M‐IGGΚ BP‐HRP were purchased from bio‐strategy delivering technologies. Anti‐GAPDH (14C10) Rabbit mAb, Anti‐rabbit IgG, and HRP‐linked Antibodies were purchased from Cell Signalling Technology. FBS, cell culture medium (DMEM and RPMI), penicillin/streptomycin (P/S) antibiotics, and Lipofectamine 2000 were purchased from Invitrogen (by Thermo Fisher Scientific). Heparin sodium salt (from Porcine Intestinal Mucosa; H4784, = 202 USP units mg^−1^), sodium dodecyl sulfate (SDS), tris‐buffered saline (TBS), 3‐(4,5‐Dimethyl‐2‐thiazolyl)‐2,5‐diphenyl‐2H‐tetrazolium bromide (MTT, 98%), and sodium TPP (85%) were purchased from Sigma‐Aldrich. Ultrapure water (18.2 MΩ cm) was obtained from a PureLab flex system and was used throughout except for siRNA‐loaded NP fabrication where nuclease (RNase) free water (DEPC‐treated) purchased from Fisher Bioreagents was used.

### Fabrication and Evaluation of Twist1‐siRNA Loaded NPs

Twist1‐siRNA‐loaded NPs were fabricated using RNase‐free water. Briefly, PEGylated polymers were dissolved in 0.1 m HCl overnight with a final concentration of 0.5 mg mL^−1^. The pH of the polymer solutions was adjusted to 5.5 using 0.1 m NaOH. Twist1‐siRNA‐loaded NPs were fabricated with TPP as a crosslinker. The twist1‐siRNA solution (40 µL, 0.2 mg mL^−1^) was mixed with TPP solution (0.335 or 0.2 mL for mPEG‐CHI and ALD‐PEG‐CHI, respectively, 0.2 mg mL^−1^, pH 5.5 acetate buffer) before being added to the polymer solution (0.4 mL), mixed quickly by pipetting and then vortexed for one min. These NPs had a N/P ratio of 50 and are referred to as mPEG‐CHI‐siRNA and ALD‐PEG‐CHI‐siRNA NPs. The fabricated NPs were filtered through a 30 kDa Amicon centrifugal filter at 3000 × g (4400 rpm) (Eppendorf, 5702) for 10 min to remove any free TPP and unencapsulated twist1‐siRNA. The concentrated NPs suspension was collected and used for different assays. The encapsulation efficiency of siRNA in mPEG‐CHI and ALD‐PEG‐CHI NPs was evaluated by running the siRNA‐loaded NPs on a 4% agarose gel containing GelRed as previously described.^[^ ^34^ ^]^ A Nanosizer (ZEN3600, MALVERN Instruments) was used to measure the z‐average, number weighted mean, polydispersity index (PDI), and ζ‐potential of the NPs as previously described.^[^ ^34^ ^]^

### Serum Protection Assay

Serum protection of twist1‐siRNA by the NPs was evaluated by a serum protection assay.^[^ ^36^ ^]^ Briefly, a 200 µL volume of twist1‐siRNA loaded NPs (4 µg siRNA) was mixed with the same volume of FBS and placed at 37 °C in the shaker (100 rpm) water bath. The naked twist1‐siRNA treated in the same manner was used as a control. Sample aliquots (40 µL) were collected at 0, 1, 2, 4, 6, 21, and 30 h time points and stored at −20 °C for further use. Before electrophoresis, samples were placed in a water bath at 60 °C for 3 min to terminate the serum activity. Then, 5 µL of heparin (1000 U mL^−1^) was added to each sample for a displacement of encapsulated twist1‐siRNA from the NPs. The integrity of the displaced twist1‐siRNA was evaluated by performing electrophoresis on a 4% agarose gel containing GelRed and gel visualization. Serum protection was evaluated in triplicate (*n* = 3).

### Cell Culture

The passage number for the two cell lines was <25 for different experiments, however, most experiments were performed using cell passage between 10 and 20. The cells were grown in T‐25 flasks with 5% CO_2_ and 90% relative humidity in a 37 °C incubator. The MDA‐MB‐231 cells were cultured in DMEM supplemented with 10% FBS and 1% P/S antibiotics while 4T1 cells were cultured in RPMI medium supplemented with 10% FBS and 1% P/S antibiotics. The cells were sub‐cultured every 3–4 days (>80% confluency) with a split ratio of 10–20:1.

### MTT Assay

The cytotoxicity was evaluated by an MTT assay as previously reported.^[^ ^32^ ^]^ Cells were seeded in 96 well plates at a seeding density of 3000 cells per well in 100 µL DMEM or RPMI culture medium. After 24 h, the spent media was aspirated, and cells were treated with different samples for 48 h. The samples included twist1‐siRNA loaded NPs (3.12–50 nm twist1‐siRNA equivalent) and positive control lipofectamine (twist1‐siRNA 3–80 nm). After 48 h of treatment, the MTT assay was performed in triplicate (*n* = 3), and the cell viability was evaluated according to the ISO 10993–5 standard.

### Wound Healing Assay

The in vitro wound healing assay of twist1‐siRNA loaded NPs was evaluated against 4T1 cells as previously described.^[^ ^29^, ^53^, ^58^ ^]^ The assay was performed in 12‐well plates (1 × 10^5^ cells well^−1^). The cells were incubated at 37 °C in a 5% CO_2_ incubator for 24 h after which a sterile 200 µL tip was used to create a scratch line on the monolayer of the cells. The media was aspirated, and cells were washed with PBS. The NPs diluted in 10% FBS and antibiotic‐free RPMI culture media were added to cells at concentrations of 5, 10, and 50 nm equivalents. After 4–6 h, a cell culture medium containing 10% FBS and 1% P/S antibiotics was added. The control cells were treated in the same manner without adding any NPs. At different time points, the images of the scratch area were captured using a microscope (OLYMPUS IX51). This assay was done in triplicates (*n* = 3).

### Western Blot

MDA‐MB‐231 and 4T1 cells were seeded at 2.5 × 10^5^ cells well^−1^ in 6‐well plates and treated with NP suspensions (50 nm equivalent of twist1‐siRNA). Cells were harvested by adding 150 µL SDS lysis buffer after carefully removing the cell culture media. After lysis, samples were stored at −20° C until further use. The protein samples were run on a 10‐well NuPAGE 4–12% bis‐tris gradient gel with MES buffer. The gel was run at 40–45 mA constant current for 90 min. A piece (9 × 7 cm) of millipore immobilon membrane (Cat#IPVH00010) was used for transfer. After washing, the membrane was blocked with 20 mL 5% blotto (5% skim milk powder in 1×TBS) in a Sterilin dish for ≈30 min on a rocking platform mixer. Anti‐TWIST1 antibody (Twist2C1: sc‐81417) was diluted in 4 mL of 5% blotto with 0.05% Tween‐20 (TBS‐T) at 1:250 dilution and added to blot at 4 °C overnight. After washing, control anti‐GAPDH or anti‐β‐Actin antibodies were treated similarly but a 1:1000 dilution was used. The secondary HRP‐conjugated antibody was diluted in TBS‐T at a dilution of 1:2000 and added to the blot for 2 h at room temperature. After washing, the blot was exposed to the ECL reagent. The membrane was exposed in an Amersham AI600 imager to capture images. This was done in triplicates (*n* = 3).

### Statistical Analysis

The data was collected with *n*‐values as indicated for each experiment above. Data were analyzed by GraphPad prism software (9.0.0(121)). The results were represented as mean ± standard deviation and a *p*‐value of <0.05 was considered significant.

## Conflict of Interest

The authors declare no conflict of interest.

## Supporting information



Supporting Information

## Data Availability

The data that support the findings of this study are available from the corresponding author upon reasonable request.
