# Supplementary material for: Chitosan‐Based Nanoparticles for Twist1 Knockdown in 4T1 Cells
Source: Macromol Biosci. 2025 Apr 10;25(7):2400627. doi: 10.1002/mabi.202400627 (PMC12259407; doi:10.1002/mabi.202400627)
Supplement: Supplementary file 1 — Supporting Information [file MABI-25-2400627-s001.docx]

**Supplimentray Information**

**Chitosan-based nanoparticles for twist1 knockdown in 4T1 cells**

Asim Mushtaq^a^, Li Li^b^, Anitha A^a^, Lisbeth Grøndahl^a,b^***

*^a^ School of Chemistry and Molecular Biosciences, The University of Queensland, Cooper Road, Brisbane, Queensland 4072, Australia*

*^b^ Australian Institute for Bioengineering and Nanotechnology, The University of Queensland, Corner of College and Cooper Road, Brisbane, Queensland 4072, Australia*


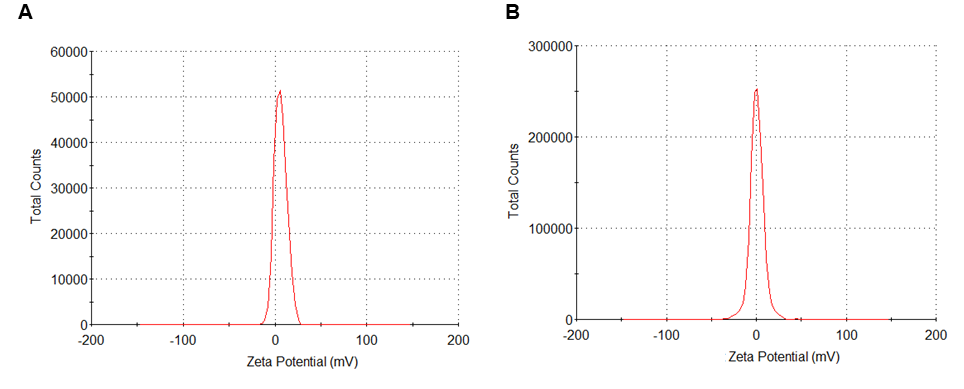


**Figure S1.** Zeta potential distribution plots for A) mPEG-CHI-siRNA NPs and B) ALD-PEG-CHI-siRNA NPs, demonstrating that these NPs have a near zero ζ-potential.


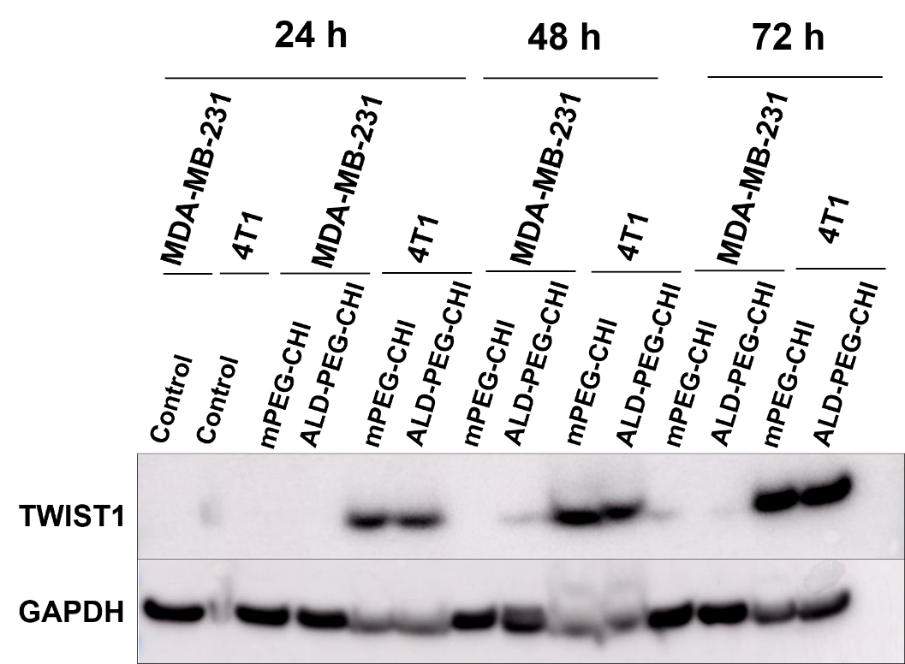


**Figure S2.** Western blot analysis of mPEG-CHI-siRNA and ALD-PEG-CHI-siRNA NPs treated 4T1 and MDA-MB-231 cells at different points. No control band was observed for twist1 (4T1 cells) due to an error in sample loading (n=1). Subsequent analysis (Figure 4 in main manuscript) showed this band. Concentration of siRNA in these NPs was 50 nM.


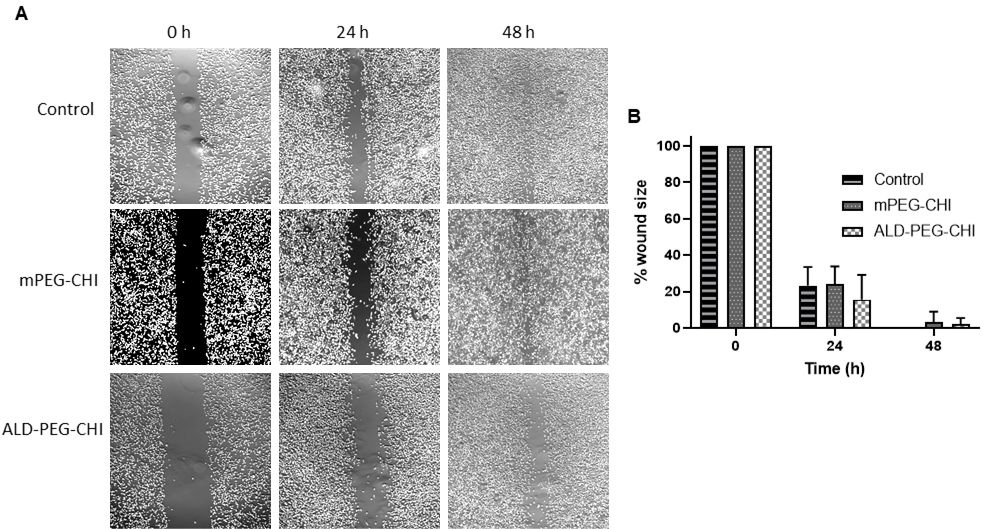


**Figure S3.** A) Representative bright field images of wound closure effect of twist1-siRNA loaded mPEG-CHI NPs and ALD-PEG-CHI NPs against MDA-MB-231 cells. B) Wound healing effect of the NP systems show no significant difference compared to control with no NPs added.


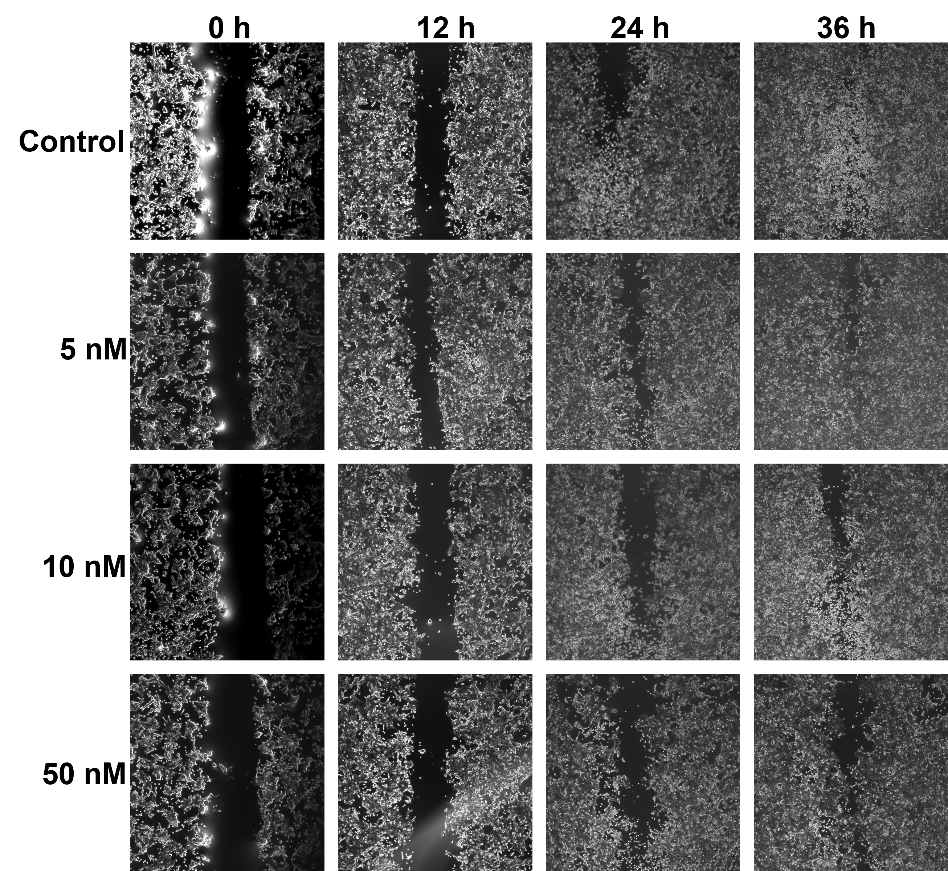


**Figure S4**. Representative bright field images of wound closure effect of twist1-siRNA loaded mPEG-CHI NPs against 4T1 cells. The images were captured using 4x magnification. Seeding density = 2.8 × 10^4^ cells/cm^2^, n = 3, images show one representative repeat.


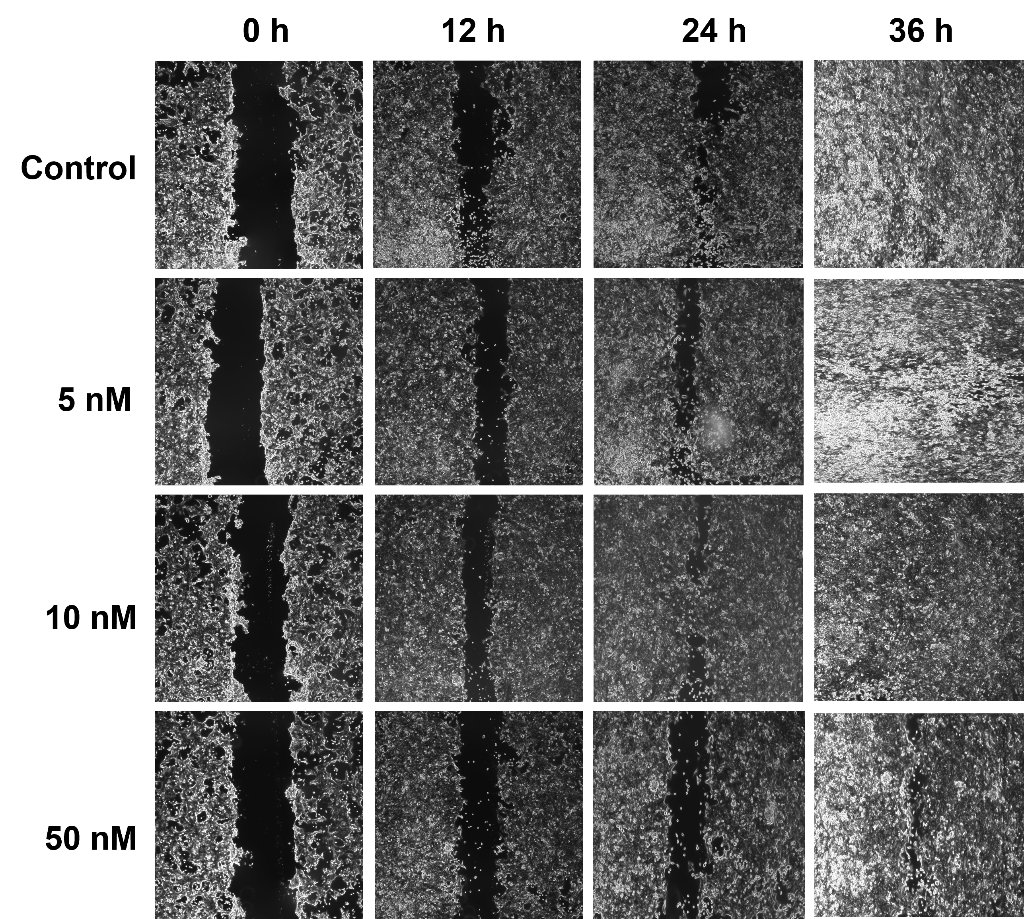


**Figure S5**. Representative bright field images of wound closure effect of twist1-siRNA loaded ALD-PEG-CHI NPs against 4T1 cells. The images were captured using 4x magnification, Seeding density = 3.3 × 10^4^ cells/cm^2^, n = 3, images show one representative repeat.

**
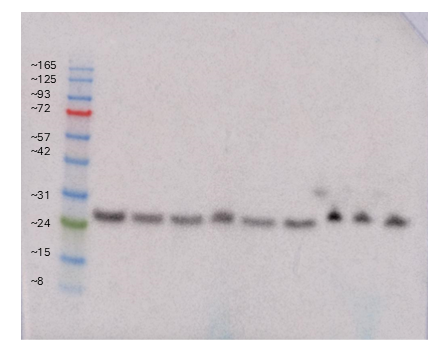
**

**Figure S6.** Western blot analysis of twist1-siRNA used in the current study. Markers confirm the expected size of 26 kDa and high purity.
